# Male Killing *Spiroplasma* Preferentially Disrupts Neural Development in the *Drosophila melanogaster* Embryo

**DOI:** 10.1371/journal.pone.0079368

**Published:** 2013-11-13

**Authors:** Jennifer Martin, Trisha Chong, Patrick M. Ferree

**Affiliations:** W. M. Keck Science Department, Claremont McKenna, Scripps, and Pitzer Colleges, Claremont, California, United States of America; Ecole Normale Supérieur de Lyon, France

## Abstract

Male killing bacteria such as *Spiroplasma* are widespread pathogens of numerous arthropods including *Drosophila melanogaster*. These maternally transmitted bacteria can bias host sex ratios toward the female sex in order to ‘selfishly’ enhance bacterial transmission. However, little is known about the specific means by which these pathogens disrupt host development in order to kill males. Here we show that a male-killing *Spiroplasma* strain severely disrupts nervous tissue development in male but not female *D. melanogaster* embryos. The neuroblasts, or neuron progenitors, form properly and their daughter cells differentiate into neurons of the ventral nerve chord. However, the neurons fail to pack together properly and they produce highly abnormal axons. In contrast, non-neural tissue, such as mesoderm, and body segmentation appear normal during this time, although the entire male embryo becomes highly abnormal during later stages. Finally, we found that *Spiroplasma* is altogether absent from the neural tissue but localizes within the gut and the epithelium immediately surrounding the neural tissue, suggesting that the bacterium secretes a toxin that affects neural tissue development across tissue boundaries. Together these findings demonstrate the unique ability of this insect pathogen to preferentially affect development of a specific embryonic tissue to induce male killing.

## Introduction

A hallmark of higher eukaryotes is their association with a wide variety of bacterial symbionts. Many such bacteria live commensally or even mutualistically within host tissues. However, some bacteria can negatively alter host cellular and developmental processes in order to achieve enhanced bacterial transmission. A primary example is the bacteria-induced killing of male progeny during their development, a phenomenon that is widespread among insect species [1]. Certain male-killing strains of *Spiroplasma*, which are spiral-shaped and cell wall-less bacteria belonging to the Mollicute class [2], infect multiple species within the *Drosophila* (*i.e*., fruit fly) genus [3]. A wide range of species outside of *Drosophila*, including butterflies, ladybird beetles, and wasps, also are infected by these and other male killing pathogens [4]–[9]. Male killing can dramatically bias the sex ratios of host populations toward the female sex, an effect that is believed to benefit the male-killing bacteria because they are transmitted solely from infected females to their offspring. Male progeny, in contrast, are ‘dead ends’ for bacterial transmission. Moreover, in some of these host-pathogen systems, the killing of male progeny may provide advantages, such as increased access to food and perhaps other important resources to host females, thereby enhancing bacterial spread [1]. The occurrence of male killing poses unique challenges to understanding how such effects influence host population dynamics and, ultimately, the evolution of host and pathogen.

A fundamental aspect of male killing is how the causal bacteria target males for death. This issue involves understanding male killing effects at both the molecular and developmental levels. Regarding the molecular level, two male-specific processes, somatic sex determination and dosage compensation, have been previously tested as possible targets of male embryonic lethality in the genetic model organism, *Drosophila melanogaster*. Females infected with a male-killing strain of *Spiroplasma* were transformed into intersex males through mutant alleles of the *doublesex* (*dsx*) and *transformer* (*tra*) genes [10]. These individuals when infected were fully viable, suggesting that somatic sexual identity is not involved in male killing by this bacterium [10]. In a different study also conducted in *D. melanogaster*, the dosage compensation pathway was genetically tested as a potential target of male killing. The level of male killing was assessed in the genetic background of strong loss-of-function mutations for five different genes, each encoding a protein component of the Dosage Compensation Complex (DCC) [11]. This complex is required to generate a two-fold transcriptional increase of most genes on the single X chromosome in males in order to match gene expression levels found in females, which have two X chromosomes [12]. Mutations in each of these five genes partially suppressed male embryonic lethality caused by a male-killing *Spiroplasma* strain that normally infects *Drosophila nebulosa* [11]. Together, these experiments suggest that some aspect(s) of the male-specific dosage compensation pathway, and not maleness of somatic cells, is the molecular target of male killing.

The developmental basis of male killing has been addressed through several studies involving different bacterial strains and host species. *D. nebulosa* male embryos infected with a native male-killing *Spiroplasma* strain known as NSRO (for *nebulosa* sex ratio organism) exhibited abnormal morphology, such as amorphous internal structures and a lack of external segmentation, during gastrulation [13]. *Spiroplasma*-infected *D. willistoni* embryos showed similar defects during gastrulation but also much earlier ones, including improperly formed meiotic products shortly after egg laying, and multi-polar spindles and asynchronously dividing nuclei during the mitotic cleavage divisions [14]. The presence of mitotic defects during cleavage suggests either that *Spiroplasma* induces these defects specifically by targeting some unknown male-specific cell cycle-related process during the earliest stages of embryogenesis or, alternatively, the bacterium affects these mitotic divisions in a sex-independent manner while also inducing a different specific male-killing effect during later embryogenesis. Additionally, infected *D. nebulosa* males exhibited heightened levels of nuclei containing fragmented DNA during mid-gastrulation, suggesting that *Spiroplasma* may either directly or indirectly induce apoptotic pathways or non-specific cellular decay [13]. Taken together, these studies imply that (*i*) male killing involves several distinct cellular and developmental phenotypes depending on the host species and bacterial strain and (*ii*) these phenotypes may derive from defects that are either specific or non-specific to the male sex.

An additional, important issue pertaining to the developmental basis of male killing is whether *Spiroplasma* preferentially affects certain tissues during the onset of the embryonic lethal phase. This issue was previously addressed indirectly through two different studies performed in *D. melanogaster*. Specific mutations that cause high levels of chromosomal non-disjunction were used to produce *Spiroplasma*-infected XX females that were mosaic for regions of XO (*i.e*., male) cells [15]. Fate mapping of these individuals revealed that XO cells were present in all areas of the blastula except for regions destined to become the muscle and nervous tissues [15]. This result suggested that *Spiroplasma* specifically targets one or both of these tissues during embryogenesis. In order to distinguish between these possibilities, a subsequent study examined the developmental trajectory of cells in tissue culture that had been dissociated from undifferentiated embryos infected with *Spiroplasma* [16]. Very few neuron-like cells were observed to differentiate from these dissociated embryonic cells compared to other tissue types, whereas muscle-like cells appeared in relatively higher numbers but were less abundant than those arising in the absence of *Spiroplasma* [16]. While these studies support the idea that *Spiroplasma* may specifically affect the neural tissues, they leave open the possibility that non-neural tissues also may be affected at the onset of male killing. Identifying the specific tissue(s) that are affected, the nature of the cellular defects involved, and precisely when during development the onset of male killing occurs will be important for further molecular mechanistic studies and for obtaining a more complete understanding of this phenomenon.

We have performed cytological analyses of *Spiroplasma*-infected *D. melanogaster* embryos in order to investigate the developmental basis of male embryonic lethality in this system. We discovered that the earliest visible defect is a strikingly abnormal central nervous system (CNS) during mid-embryogenesis. Specifically, we found that the neurons differentiate but fail to properly pack into the ventral nerve chord and are inhibited from forming normal axons. The cellular morphology of the mesoderm, the tissue that gives rise to muscle, and embryo segmentation appears normal at this developmental time, strongly suggesting that the neural primordial tissue is preferentially affected at the onset of male killing. Finally, we found that *Spiroplasma* is altogether absent from the neural tissue while localizing within the gut and other non-neural tissues. Together these findings have important implications for how *Spiroplasma* may kill males at the molecular level while preferentially affecting specific tissues.

## Results

### MSRO *Spiroplasma* Preferentially Disrupts Neural Development at the Onset of Male Killing

In order to address the developmental basis of *Spiroplasma*-induced male killing in *D. melanogaster*, we employed experiments using the wild type *D. melanogaster* line, Canton-S. This fly line was infected by introgression with the male-killing *Spiroplasma* strain MSRO (for *melanogaster* sex ratio organism), which is native to this species (see Methods) [17]. Infected females of this line consistently produced broods that are extremely biased toward the female sex. The percentage of female progeny per brood ranged between ∼99–100% for more than 40 generations of propagation. Thus, this *Spiroplasma*-infected Canton-S fly strain exhibited a strong make killing phenotype that persisted from generation to generation in the laboratory.

**Figure 1:**
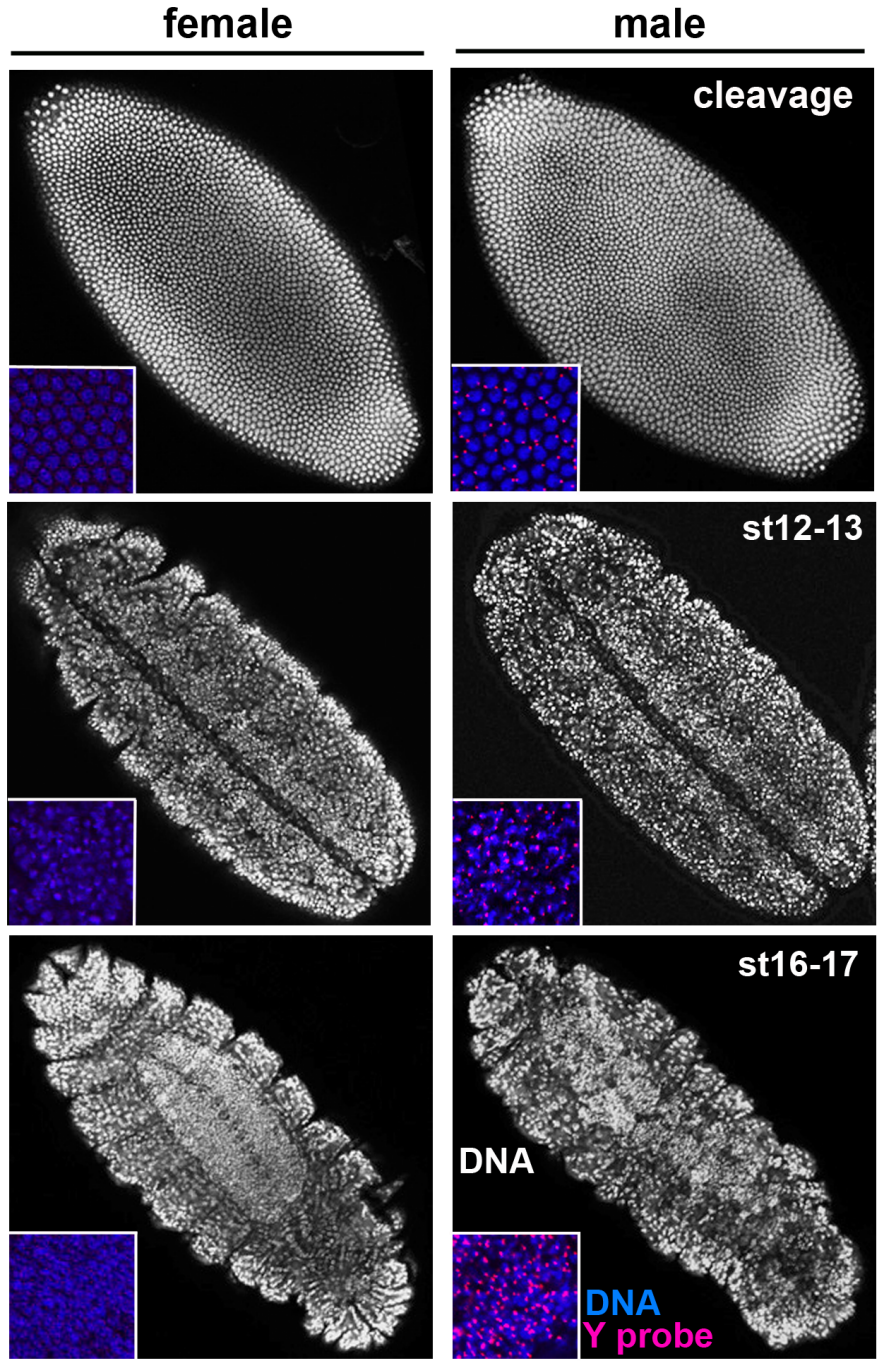
*Spiroplasma* alters general morphology of the whole male during late embryogenesis. All embryos shown are MSRO-infected. Insets depict DNA (blue) and a Y-chromosome specific probe (red), which indicates sex of the embryo.

We first examined infected embryos undergoing mitotic cleavage divisions. DNA fluorescence *in situ* hybridization (FISH) with a probe that recognizes repetitive sequences on the Y chromosome was used to unambiguously distinguish male from female embryos. All infected 0–2.5 hr-old male and female embryos (n = 20 and 23, respectively) exhibited evenly spaced nuclei that divided synchronously during cleavage (Figure 1) in a manner that was indistinguishable from uninfected embryos (not shown). Thus, MSRO *Spiroplasma* does not induce early mitotic defects in *D. melanogaster* like those that are caused by other *Spiroplasma* strains in different Drosophila species or by a male-killing *Wolbachia* strain in *D. melanogaster* [9], [14]. Infected 8–10 hr-old (stage 12–13) male and female embryos also appeared indistinguishable from one another at the morphological level (Figure 1). In both sexes, overall embryo shape and cell density in the outer (epithelial) layer appeared normal. However, in late stages (18+ hours after egg laying), male embryos became severely misshapen and contained large regions of irregularly spaced cells across the whole animal (Figure 1). These irregularities became more pronounced during subsequent developmental stages (Figure 1). In contrast, infected female embryos showed no such cellular or other morphological defects (Figure 1).

**Figure 2:**
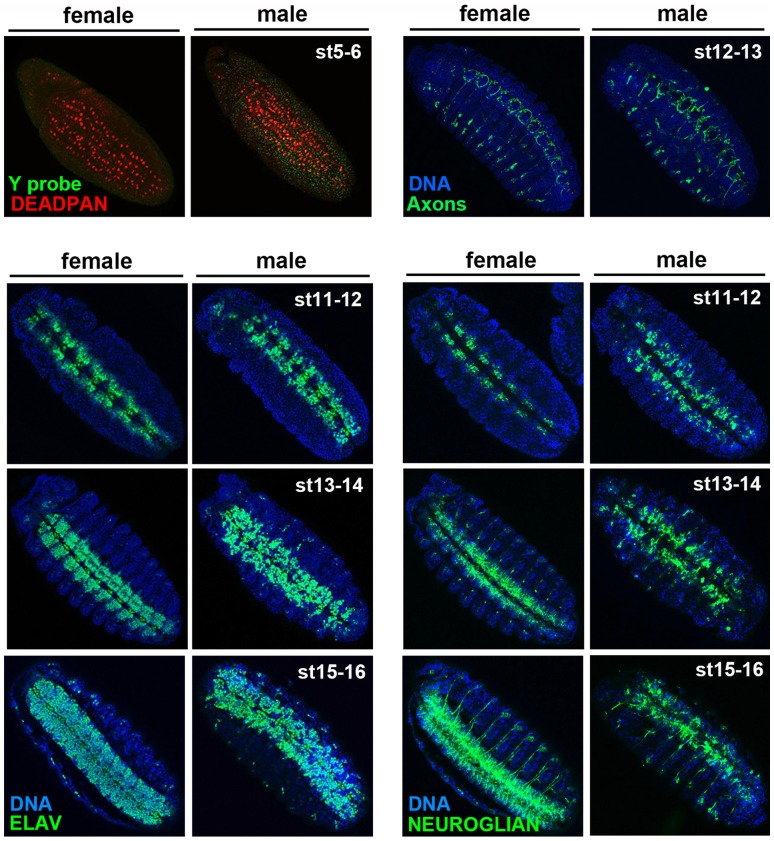
*Spiroplasma*-infected male embryos exhibit normal neuroblasts but highly irregular neurons. All embryos shown are MSRO-infected. The neuroblasts are highlighted by anti-Deadpan (red in top left panels); the neuron cell bodies are marked by anti-Elav (green in bottom left panels); the neuron axons are marked by the 22C10 antibody (green in top right panels); and neurons, glial cells, and axons are indicated by anti-Neuroglian (green in bottom right panels).

Given the results of previous studies suggesting that neural tissue may be preferentially affected by *Spiroplasma*, we directly visualized the tissue of the central nervous system (CNS) as it developed in infected embryos. Normally, formation of the CNS begins through the specification of neuroblasts, the neuronal progenitors, from ectodermal cells along the ventral midline during stage 9 of embryogenesis (∼4 hr after egg laying) (for a complete review of embryonic neural development in Drosophila, see [18], [19]). Once specified, these neuroblasts delaminate from the ectodermal layer and move a short distance inside this layer. Subsequently these neuroblasts divide multiple times, giving rise to populations of daughter neurons and glial cells that are arranged in clusters bilaterally along the midline within each segment. By stage 11 (∼6 hrs) many of these cells strongly express certain proteins including Elav and Neuroglian [20]–[22]. During stage 13 (∼9.5–10.5 hrs), the cells of the central nervous system form a wide, bilaterally symmetrical stripe along the ventral midline, referred to as the ventral nerve chord, as these cells become more numerous. A subset of neurons form axons that reach dorso-laterally in each embryo segment, while others produce axons that run longitudinally along the midline or cross the midline to connect the two sides of CNS. By stage 14 (∼11 hrs), the ventral nerve chord becomes visibly condensed. These landmark events of early CNS development appeared normal in infected female embryos (n = 42; Figure 2) and in uninfected embryos of both sexes (not shown).

In *Spiroplasma*-infected male embryos (n = 6), the neuroblasts formed in a manner that is indistinguishable from infected female embryos (n = 5) (Figure 2). Additionally, clusters of neuroblast daughters formed along the ventral midline during stage 11 (Figure 2). These cells were highlighted brightly by both anti-Elav and anti-Neuroglian antibodies, indicating that they appropriately undergo differentiation into neurons. However, these clusters appeared disorganized very early in their formation specifically in male embryos (Figure 2), and they became more disorganized during further cell division (Figure 2). Specifically, the bodies of these cells, marked by anti-Elav which stains the neuronal nuclei, appeared loosely organized in all infected male embryos (n = 16), in contrast to the tightly packed arrangement of neuronal bodies in the female CNS (Figure 2). Additionally, all stage 12–14 male embryos exhibited highly abnormal axons, as seen with anti-Neuroglian (n = 19) and another axon-specific antibody (Figure 2). This latter antibody also highlights the axons of the peripheral neurons [23]; these axons also appeared disorganized (Figure 2), suggesting that *Spiroplasma* affects all embryonic neural tissue. Along most of the ventral nerve chord, we observed few or no axons, while in some isolated regions, these structures formed but they were highly abnormal, appearing to follow irregular paths within each segment (Figure 2). Additionally, the two sides of the CNS failed to become properly connected due to a lack of axons (Figure 2). Despite these abnormalities, the overall size of the ventral nerve chord appeared to be approximately the same in infected male and female embryos, as well as in uninfected male embryos (Figure 2), indirectly suggesting that cell proliferation is not disrupted.

**Figure 3:**
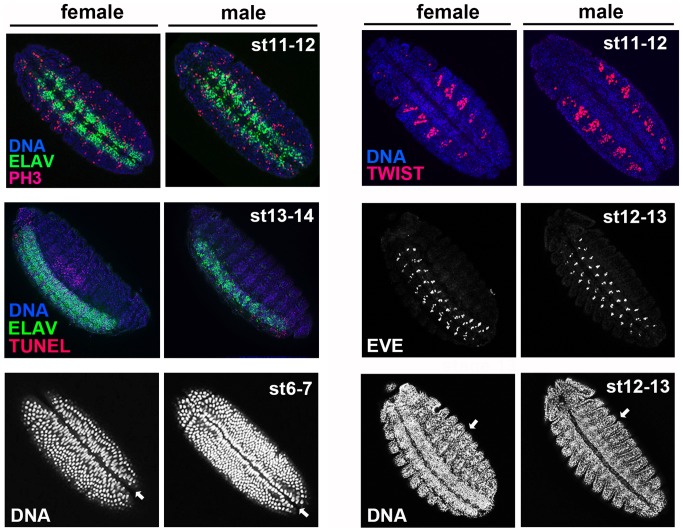
*Spiroplasma* does not cause abnormal levels of cell proliferation, cell death, or abnormal defects in non-neural tissues or other processes during the onset of neural defects. All embryos shown are MSRO-infected. Cell proliferation is depicted by anti-phospho histone H3 (PH3) (red in top left panels); broken DNA, an indicator of cell death, is shown by TUNEL (red in middle left panels); The ventral midline invagination can be seen with DNA staining (indicated by white arrows in bottom left panels); The developing mesoderm is highlighted by anti-Twist (red in top right panels); A subset of motoneurons located in odd body segments is marked by anti-even-skipped (white in middle right panels); external body segments are seen through DNA staining (indicated by white arrows in bottom right panels).

In order to more directly address neuronal proliferation in infected male embryos, and to ascertain if the male neural tissue defects involve cell death, we stained embryos for phosphorylated Histone H3 (PH3), a mitotic marker, and for broken DNA by using terminal deoxynucleotidyl transferase dUTP nick end labeling (TUNEL), respectively. *Spiroplasma*-infected male and female embryos showed similar levels of PH3-positive cells across the CNS, which is where the majority of cell division occurs during mid-embryogenesis (Figure 3). Additionally, no heightened levels of nuclei with broken DNA, an indicator of cell death, were observed in infected male embryos, when compared to female embryos (Figure 3). Thus, the neural tissue defects in male embryos caused by *Spiroplasma* likely do not involve abnormal cell proliferation or death.

### *Spiroplasma* does not Affect Non-neural Tissues or Segmentation during the Onset of Neural Tissue Defects

In order to test if *Spiroplasma* primarily affects the developing neural tissue during the onset of male killing, we examined non-neural tissues and other developmental processes in infected embryos. We first visualized formation of the ventral furrow, which is produced by invagination of ventral epithelial cells following cellularization in order to produce the mesoderm. The ventral furrow appeared normal in infected stage 6–7 (3–3.5 hr) embryos of both sexes (Figure 3). Additionally, we assessed early mesoderm morphology by staining this tissue with an anti-Twist antibody. During stages 11–12 when the neural defects become apparent, the segmented regions of mesoderm running laterally on either side of the CNS were indistinguishable between both sexes (Figure 3). Finally, we stained infected embryos with an antibody against the protein Even-skipped (Eve), which first appears around cellularization in distinct bands that mark odd-numbered segments along the anterior-posterior axis [24]. However, Eve also is expressed during CNS establishment in distinct motoneurons that are positioned as bands in the body segments [25]. We found that bands of Eve-positive neurons appeared normal in both infected male and female embryos despite that the overall CNS morphology is abnormal at this time (Figure 3). This finding suggests either that *Spiroplasma* may preferentially affect certain subsets of neurons and not others, or it may instead reflect the limited ability of a single, stained neuron subset to reflect overall CNS disorganization caused by *Spiroplasma*. Nevertheless, this normal Eve pattern in infected male embryos is consistent with the fact that these individuals also showed well-defined external segmentation during the same developmental time (Figure 3). Together these findings strongly suggest that the onset of male killing involves disruption of neural tissue development but not non-neural tissues or segmentation, while the entire male embryo becomes defective during later stages (Figure 1).

### *Spiroplasma* Bacteria are Completely Absent from the Neural Tissue

**Figure 4:**
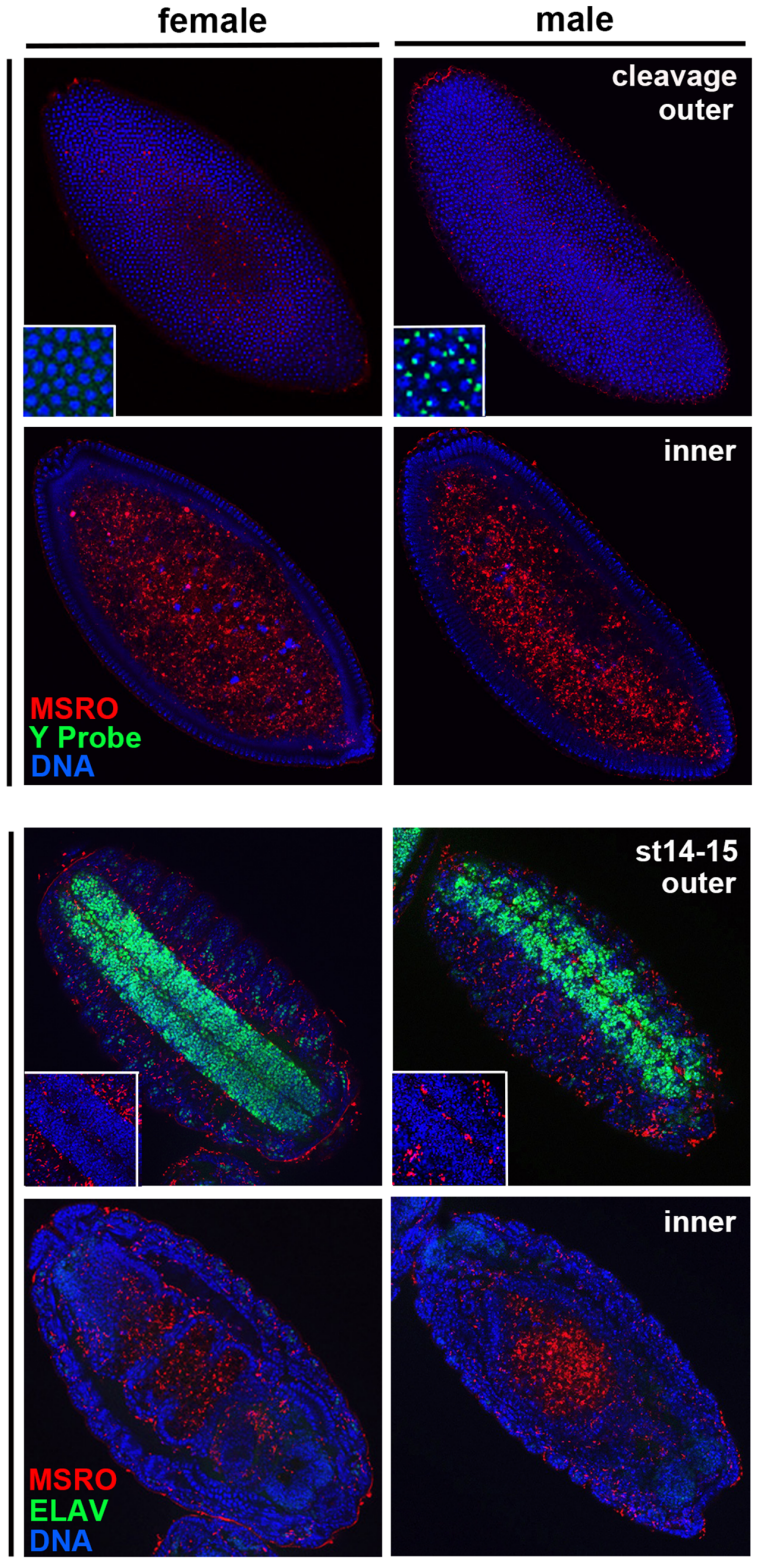
*Spiroplasma* is completely absent from the neural tissue but heavily concentrates in other tissues of both male and female embryos. High levels of bacteria (red) are located in the yolky interior of the cleavage blastoderm, while few bacteria can be seen around the cells of the epithelium (top panels). During later embryonic stages, no bacteria can be seen to overlap with the cells of the ventral nerve chord (insets in the bottom panels). During this time high levels of *Spiroplasma* are present both inside and outside the gut (bottom panels).

We sought to determine if MSRO *Spiroplasma* preferentially localize within the neural tissue, a pattern that could explain how the bacteria are capable of specifically affecting this tissue during mid stages. To do this, we stained infected embryos with an antibody raised against MSRO *Spiroplasma* [26]. This antibody highlighted bright cytoplasmic bodies in *Spiroplasma*-infected embryos (Figure 4) but not in uninfected embryos (not shown). These cytoplasmic bodies are highly similar to those previously observed in MSRO-infected oocytes [26], thus strongly arguing that they are individual *Spiroplasma*. In late cleavage embryos, the bacteria were sparsely present in the outer epithelial layer but were very dense within the yolky interior (Figure 4). In stage 14–15 embryos, *Spiroplasma* continued to be enriched in the interior of the embryo, especially within and around the gut lumen (Figure 4). However, interestingly, the bacteria were completely absent from the neural tissue but localized at low levels in the surrounding tissues (Figure 4).

### Rare Surviving Males Contain Little or no *Spiroplasma*

In our experiments we observed rare third instar male larvae and adults (<0.1% of all progeny) that were produced from infected females. We examined the escaper male larvae to investigate if *Spiroplasma* affects the development of their neural or other tissues. Infected males exhibited similar sized brains compared to those of infected females and uninfected males (Figure S1). Additionally, proliferation of neural cells in infected males was normal as indicated by similar amounts of cells undergoing mitosis (Figure S1). Non-neural tissues, including salivary glands and testes, and overall body size of infected males also were similar to control tissues (Figure S1). We then assessed the infection status of escaper male larvae by PCR amplification of the *Spiroplasma*-specific *SpoT* locus. These individuals showed very low or no *SpoT* product compared to their female siblings (Figure S2). This finding suggests that male larvae may escape the killing effect because they fail to inherit a sufficient level of bacteria. Because of this pattern our analyses do not allow us to determine if the neural tissue of male larvae is also sensitive to the male killing effect. However, we can conclude that the primary lethal phase caused by the MSRO strain is embryonic and initiates through defects arising in the early neural tissue.

## Discussion

An important step toward understanding the molecular basis of *Spiroplasma*-induced male killing is to discern precisely when and how the causal bacteria alter host development. Previous work strongly suggests that some aspect of the male-specific dosage compensation pathway is involved in this phenomenon [11]. On the practical level, knowledge of which tissue(s) are specifically targeted will greatly facilitate future mechanistic studies, such as determining the behavior of the dosage compensation machinery at the chromosomal level, by allowing directed analyses in affected regions and at the correct developmental time. Additionally, the cellular phenotypes in the affected tissues can provide important clues regarding the molecular mechanism of male killing.

In this study we sought to identify specific developmental defects that underlie male embryonic lethality caused by the MSRO strain of *Spiroplasma* in *D. melanogaster*. This knowledge is particularly important because of the promise that this genetic and genomic model organism holds for future mechanistic studies of male killing. Our results strongly suggest that, like in other *Spiroplasma* strain-host species combinations [13], [14], [27], [28], the primary lethal phase of male killing in *D. melanogaster* occurs during embryogenesis. Male embryos exhibit severe morphological defects in cellular organization across the entire embryo during late stages (18+ hrs), which likely prevents them from further development. The earliest visible defects occur during mid embryogenesis and map specifically to the newly forming neural tissue. The overall size of the early ventral nerve chord is normal in infected males. Additionally, the neurons express several neural-specific markers at their appropriate times, suggesting that their proliferation and differentiation are not affected. However, the organization of these cells becomes dramatically disrupted as they form, and they either fail to form axons or produce them in a severely irregular pattern. In contrast, other tissues and developmental processes appear normal during the onset of neural tissue defects, although the entire male becomes morphologically defective during late embryogenesis.

Why might the neural tissue primarily be affected during mid stages while multiple tissues become defective during later stages? It is possible that *Spiroplasma* secretes a toxin that induces male killing. This possibility is supported by the fact a number of bacteria are known to secrete toxins that facilitate various aspects of bacterial infection or propagation [29], [30]. In this scenario, a toxin secreted by the MSRO strain could specifically target a pathway that is unique to the neural tissue. Disruption of this tissue could lead secondarily to defects in other tissue types during later stages. Alternatively, a *Spiroplasma*-produced toxin could affect a pathway that is important for most or all tissues, but the neural tissue is affected initially because it is either more dependent on this pathway or it utilizes the pathway at an earlier developmental time than other tissues. Such a toxin would likely operate across tissue boundaries because *Spiroplasma* fail to concentrate in or immediately around the neural tissue but, instead, are highly abundant in the gut. This lack of *Spiroplasma* within the neural tissue is also found in female embryos, a factor that may help to insure that the bacteria do not indirectly disrupt development of this important tissue in the transmitting sex. Our current experiments do not allow us to assess whether *Spiroplasma* are located within the epithelial and gut cells in addition to the interstitial spaces between cells within a given tissue. However, the possibility of bacterial localization both within and outside of these cells is consistent with previous observations of *Spiroplasma* residing between the follicle cells surrounding the developing egg as well as within the egg, the latter being where the bacteria must migrate in order to be maternally transmitted [26].

Our findings support a plausible model that explains how male killing could involve dosage compensation, a process that is required for all male tissues, yet preferentially affect the neural tissue, which is essential for both sexes. Two previous lines of evidence suggest that male killing involves a dominant gain-of-function effect through dosage compensation. First, loss of DCC function rescues embryonic male killing by the NSRO strain in *D. melanogaster*, thus strongly arguing that male killing requires a functional dosage compensation complex [11]. Second, while both male killing in wild type males and the rescue of male killing in DCC mutants occur during embryogenesis, males with compromised dosage compensation survive through the third instar larval stage [31], [32]. Therefore, in the presence of NSRO, the DCC may act inappropriately at normally non-compensated loci including those located on the autosomes (*i.e.,* the non-sex chromosomes) during mid embryogenesis, just after the DCC is normally expressed [33]. Currently it is not known if other SRO strains like MSRO also target the dosage compensation pathway. However, if this scenario is true for MSRO, then it is intriguing to speculate that this *Spiroplasma* strain may induce male killing through ectopic dosage compensation of one or a few autosomally located genes that are required for early neural tissue development. Mis-expression of such genes would be expected to occur only in the male sex because the DCC does not form in females [33]. This idea is consistent with our observation that females do not exhibit any neural or other tissue-specific defects during embryogenesis. Interestingly, to our knowledge the neural tissue defects caused by MSRO-*Spiroplasma* do not appear similar to phenotypes caused by mutations in known neural-related genes. Thus, these bacteria-induced phenotypes may ultimately reveal new insights into Drosophila CNS development.

*Spiroplasma* causes a variety of different effects on embryogenesis that depend on the particular bacterial strain and host species. For example, in contrast to the phenotypes caused by the MSRO strain in *D. melanogaster* reported here, the NSRO strain alters male development in *D. nebulosa* at an earlier time in embryogenesis such that segmentation does not occur [13]. Additionally, the defects in this latter system involve heightened cell death [13]. Currently, little is known about the specific tissues that are initially affected in *D. nebulosa*. However, such phenotypic differences between male killing systems could reflect the ability of distinct bacterial strains to produce unique toxins that differentially affect unrelated host targets. Alternatively, it is possible that distinct bacterial strains produce similar toxins that interact with common host factors, but the concentrations or specific activities of these toxins may vary in a bacteria strain-dependent manner. Finally, differences in the severity of phenotype between male killing systems may primarily reflect interspecies divergence of the underlying host molecular targets. The preferential disruption of the male neural tissue by the MSRO strain in *D. melanogaster* will provide a foundation for future mechanistic and comparative studies in order to explore these ideas.

## Materials and Methods

### Bacterial Introgression

The *Spiroplasma* strain used in these experiments is the MSRO strain in a *D. melanogaster* line that was collected from Uganda [17]. In order to eliminate potential host background effects, we introgressed this MSRO strain into the wild type Canton-S fly line by crossing infected females with uninfected Canton-S males. This crossing scheme was conducted for over 40 generations through at least ten pair-wise crosses per generation. Progeny from individual broods that produced nearly all or all females were used for subsequent propagation. The resulting infected line is virtually identical to the uninfected Canton-S line except for the presence of *Spiroplasma*, a feature that insures that any phenotypic differences are due solely to the bacterium.

### Embryo Collection and Fixation

*Spiroplasma*-infected adult females were collected following their emergence from pupae and aged for 3–4 days before crossing *en masse* to uninfected Canton-S males. Mated females were then allowed to lay embryos on grape agar collection plates and the embryos were then aged at room temperature to the desired developmental time. Embryos were dechorionated in 50% bleach, fixed for 10 minutes in 4% paraformaldehyde/heptane, and devitellinized in methanol. Subsequently, embryos were rehydrated in a series of methanol:water solutions at ratios of 9∶1, 5∶5, and 1∶9, followed by three washes in 1xPBT (1xPBS and 0.1%Tween-20) before staining.

### Immunostaining and TUNEL

Fixed embryos were incubated in primary antibodies overnight at 4°C at the following dilutions: mouse anti-Elav (1∶50), mouse anti-Neuroglian (1∶50), mouse antibody 22C10 (1∶50), rabbit anti-PH3 (1∶200, Santa Cruz Biotech, USA), rat anti-Deadpan (1∶50), rat anti-Twist (1∶200) (a gift from E. Wieschaus), and rabbit anti-MSRO (1∶500) [26]. The anti Elav, Neuroglian, and 22C10 antibodies were purchased from the Developmental Studies Hybridoma Bank (University of Iowa, USA). Anti-mouse, anti-rat, and anti-rabbit secondary antibodies were conjugated with either Alexa555 or Alexa633 (Molecular Probes-Invitrogen, Inc., USA). TUNEL staining was performed by using the In Situ Cell Death Detection Kit, TMR red (Roche, Inc., USA).

### Fluorescent *in situ* Hybridization

The following Y chromosome-specific sequence was used as a fluorescent *in situ* hybridization probe in order to determine embryo sex: 5′-AAT ACA ATA CAA TAC AAT ACA ATA CAA TAC-3′. This sequence was commercially synthesized (IDT, Inc., USA) and 5′-end labeled with Cy3 for fluorescent detection. Whole mount embryo hybridizations were conducted as described [34]. For immuno-FISH, embryos were first stained with primary and secondary antibodies and then re-fixed in 4% paraformaldehyde for 45 minutes, washed three times in 1xPTX and introduced into 2xSSCT buffer before the probe hybridization procedure.

### Microscopy

Embryos were mounted in Vectashield mounting medium containing the DNA stain DAPI (Vector Labs, Inc., USA). Imaging was conducted on a Zeiss DMIRB confocal microscope. Constant gain and pinhole settings were used to collect all images within a given experiment. All images were processed with Adobe Photoshop CS5 version 12.

### PCR Analysis

To PCR-test for *Spiroplasma*, we used the *SpoT* locus with the *CO1* (*Cytochhrome Oxidase 1*) mitochondrial gene serving as a positive control [35]. Total DNA was extracted from individual third instar larvae or adults by using the QIAamp DNA Micro Kit (Qiagen, Inc.). PCR cycles ranged between 30 and 40 cycles.

## Supporting Information

Figure S1 **The size and morphology of neural and non-neural tissues are normal in escaper males at the third instar larval stage.**
(TIF)

Figure S2 **Escaper male larvae and adults contain little or no** ***Spiroplasma*** **.** PCR products of the *Spiroplasma*-specific *SpoT* locus and the mitochondrial gene CO1 are shown for the following individuals: control adult female (lane 1); control adult male (lane 2); infected adult female (lane 3); infected adult escaper male (lane 4); control larval female (lane 5); control larval male (lane 6); infected larval females (lanes 7–9); infected escaper larval males (lanes 10–12).
(TIF)
