# Supplementary figures and images for: Male Killing Spiroplasma Preferentially Disrupts Neural Development in the Drosophila melanogaster Embryo
Source: PLoS One. 2013 Nov 13;8(11):e79368. doi: 10.1371/journal.pone.0079368 (PMC3827344; doi:10.1371/journal.pone.0079368)

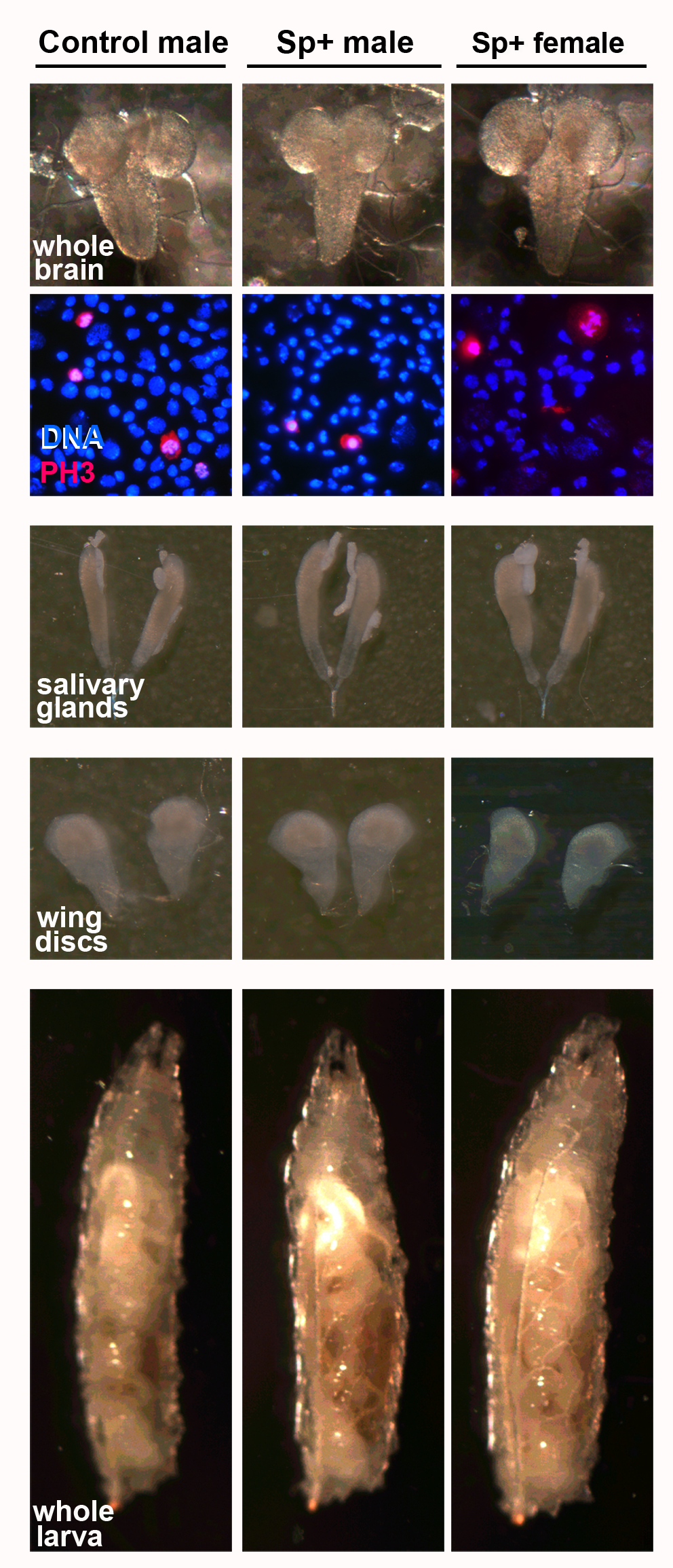

Supplement: Figure S1 — The size and morphology of neural and non-neural tissues are normal in escaper males at the third instar larval stage. (TIF) [file pone.0079368.s001.tif]

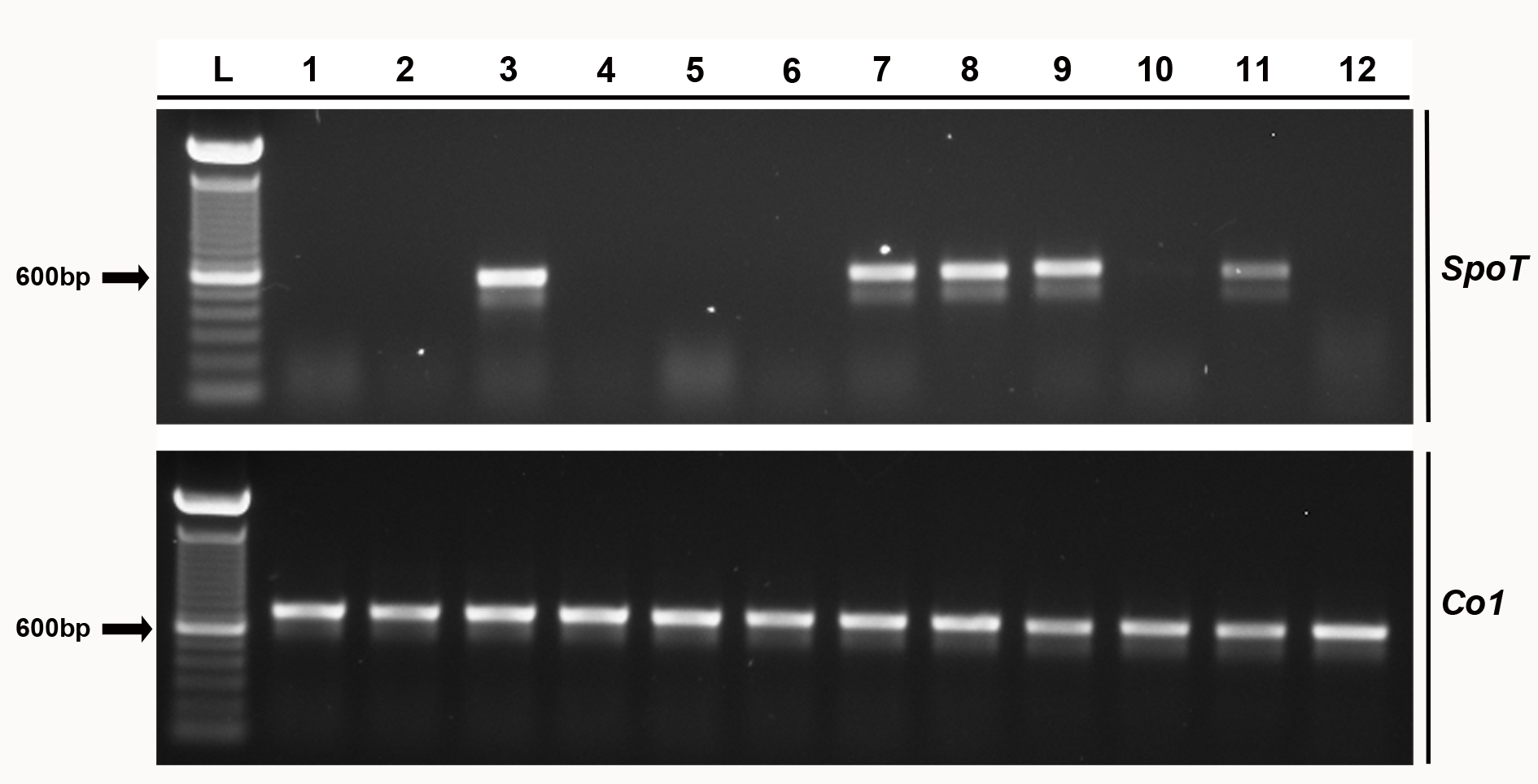

Supplement: Figure S2 — Escaper male larvae and adults contain little or no Spiroplasma . PCR products of the Spiroplasma-specific SpoT locus and the mitochondrial gene CO1 are shown for the following individuals: control adult female (lane 1); control adult male (lane 2); infected adult female (lane 3); infected adult escaper male (lane 4); control larval female (lane 5); control larval male (lane 6); infected larval females (lanes 7–9); infected escaper larval males (lanes 10–12). (TIF) [file pone.0079368.s002.tif]
